# Post-resuscitation care among emergency healthcare personnel: a cross-sectional evaluation of knowledge and clinical practices

**DOI:** 10.3389/fmed.2026.1875436

**Published:** 2026-09-04

**Authors:** Behçet Varışlı, İbrahim Uysal, Fatma Çakmak, Taygun Baykal, Hamit Çelik

**Affiliations:** 1 Department of Emergency Medicine, TC Saglik Bakanligi Bursa Sehir Hastanesi, Nilüfer, Türkiye; 2 Department of Emergency Medicine, Canakkale Onsekiz Mart Universitesi, Çanakkale, Türkiye; 3 Emergency Department, Istanbul Atlas Universitesi Tip Fakultesi, İstanbul, Türkiye; 4 Department of Neurology, Özel Buhara Hospitale, Erzurum, Türkiye

**Keywords:** ambulance, cardiac arrest, emergency healthcare personnel, knowledge assessment, post-resuscitation care

## Abstract

**Introduction:**

Cardiopulmonary resuscitation (CPR) care is a complex and dynamic process with a significant impact on mortality and morbidity. This study aimed to evaluate the knowledge levels and approaches of healthcare personnel providing emergency medical services regarding post-resuscitation care.

**Methods:**

This descriptive, cross-sectional study included 318 healthcare personnel working in emergency departments and emergency ambulances, recruited using purposive sampling. The data collection tool was developed based on the post-resuscitation care guidelines of the European Resuscitation Council (ERC), and its content validity was evaluated. Data were analyzed using descriptive statistics, *t*-tests, and analysis of variance (ANOVA), and effect sizes were calculated.

**Results:**

Participants provided advanced life support to a mean of 4.90 ± 0.29 cardiac arrest cases per month (8.65 ± 0.76 in the emergency department and 3.27 ± 0.16 in emergency medical services). Patients who achieved return of spontaneous circulation (ROSC) were transported to the emergency department in a mean of 20.35 ± 0.93 min and subsequently transferred from the emergency department to the intensive care unit in 95.26 ± 11.73 min. The mean post-CPR knowledge score was 50.08 ± 13.78 out of 100, indicating important knowledge gaps in essential post-resuscitation care practices. Emergency department personnel had significantly higher knowledge scores than EMS personnel (56.86 ± 13.84 vs. 46.87 ± 12.56; *t* = 6.401, *p* < .001). The lowest correct response rates were observed for seizure management after CPR and target blood glucose levels after cardiac arrest. Knowledge scores decreased with increasing time since the last in-service training and with increasing years of professional experience.

**Discussion:**

Professional role, clinical experience, and the recency of training appear to influence post-resuscitation care knowledge. These findings support restructuring in-service education toward repeated, practice-based assessments rather than predominantly theoretical instruction. Regular refresher training and practical educational strategies may improve knowledge retention and its translation into clinical practice.

## 1 Introduction

Cardiac arrest is a life-threatening clinical condition characterized by the sudden cessation of circulatory and respiratory functions, and its primary treatment is cardiopulmonary resuscitation (CPR) (1). The principal objective of resuscitation is to achieve return of spontaneous circulation (ROSC) while preserving meaningful neurological recovery (2). However, restoration of spontaneous circulation alone is insufficient, as post-CPR care represents a complex and challenging process that substantially influences mortality and morbidity outcomes (2). Therefore, the implementation of multidisciplinary and goal-directed post-CPR care protocols is essential to reduce early mortality and improve quality of life after cardiac arrest (3). Appropriate post-CPR care is considered one of the key determinants of survival and favorable neurological outcomes (3).

Previous studies have demonstrated that survival rates following in-hospital cardiac arrest are generally higher than those observed after out-of-hospital cardiac arrest, emphasizing the urgent need to optimize prehospital resuscitation practices and post-CPR management strategies (2, 3). Emergency healthcare personnel are responsible for the initial professional medical management of both out-of-hospital cardiac arrest cases and cardiac arrests occurring in emergency departments. Consequently, the knowledge and clinical competencies of emergency healthcare providers regarding CPR and post-CPR care are critically important for patient prognosis (3, 4).

In-hospital cardiac arrests occur in settings equipped with advanced medical technologies, continuous monitoring systems, and multidisciplinary healthcare teams. In contrast, out-of-hospital cardiac arrests are managed under more constrained conditions characterized by limited personnel capacity, unpredictable environmental factors, and time-sensitive interventions. These significant differences require varying levels of clinical decision-making skills and practical competencies among emergency healthcare professionals working in prehospital and hospital settings (5).

To improve survival rates, it is crucial to regularly assess the consistency between theoretical knowledge and practical application in accordance with current resuscitation guidelines (6). These practical applications depend largely on healthcare providers' ability to perform them correctly (7).

This study provides a comprehensive assessment of post-CPR care practices among healthcare professionals working in both prehospital emergency medical services and emergency departments in Turkey. The study aims to evaluate the level of knowledge and clinical practices regarding post-resuscitation care in light of current resuscitation guidelines. Additionally, it seeks to highlight the relationship between theoretical competencies and differences in real-world clinical practice. In this context, the study aims to provide an evidence-based and strategic framework for the standardization and improvement of post-resuscitation care in emergency medicine practice.

## 2 Materials and methods

### 2.1 Study design and participants

This descriptive cross-sectional study was conducted to evaluate the knowledge levels and clinical approaches of emergency healthcare personnel regarding post-resuscitation care. Data were collected from healthcare professionals working in emergency departments and 112 Emergency Medical Services (EMS) ambulance units, including physicians, paramedics, emergency medical technicians (EMTs), nurses, and health officers. In this study, the official job titles of the emergency healthcare personnel served as a proxy for the level of formal education required for their respective roles. Emergency medical technicians possessed high school-level vocational training; paramedics held a two-year associate degree; nurses and healthcare personnel had attained a four-year bachelor's degree; and physicians had completed a six-year medical degree. Thus, professional titles were utilized to establish the minimum educational baseline for each occupational cohort.

The study received ethical approval from the Health Sciences Ethics Committee of Çanakkale Onsekiz Mart University (Decision No. 12/27, dated February 18, 2025). Following ethical committee approval, the survey form created via Google Forms was sent to the chief physicians of provincial emergency departments and state hospitals in March 2025, along with the research permission request, and the data collection process was completed in May 2025. The online link to the survey form was sent to the emergency service and emergency ambulance personnel of the institutions that approved the research via WhatsApp groups. The survey form was limited to one answer per participant, and upon clicking the link, participants were first presented with a consent form stating that participation was voluntary and that they would participate in the survey only if they gave their consent. Data were collected from 342 participants from 17 different provinces across Turkey. After the dataset was downloaded, it was first examined for incomplete and inappropriate responses. Nine surveys containing missing data were excluded. In addition, data from 15 participants who reported never having performed resuscitation before were also removed from the dataset. The final dataset consisted of data from 318 participants.

The socio-demographic characteristics of the participants are presented in Table 1.

**Table 1:** Socio-Demographic and descriptive characteristics of participants (*n* = 318).

Variables	Groups	Total		EMS Personnel		ED Personnel	
		n	%	n	%	n	%
Gender	Female	219	68.9	132	61.1	87	85.3
	Male	99	31.1	84	38.9	15	14.7
Title	Physician (Phys)	21	6.6	-	-	21	20.6
	Paramedic	141	44.3	123	56.9	18	17.6
	Emergency Medical Technician (EMT)	105	33.0	93	43.1	12	11.8
	Other (Health Officer/Nurse)	51	16	–	–	51	50
Professional experience	0–1 Years	30	9.4	22	10.2	8	7.8
	2–5 Years	34	10.7	27	12.5	7	6.9
	6–10 Years	59	18.6	38	17.6	21	20.6
	11–20 Years	156	49.1	120	55.6	36	35.3
	21 Years and Over	39	12.3	9	4.2	30	29.4
Time has Passed Since the Last Advanced Life Support Training	Less than 1 year	87	27.4	36	16.7	51	50
	1–2 Years	75	23.6	54	25	21	20.6
	3–5 Years	102	32.1	84	38.9	18	17.6
	More than 5 years	54	17.0	42	19.4	12	11.8
Status of Following Relevant Current Guidelines	Yes	249	78.3	117	81.9	72	70.6
	No	69	21.7	39	18.1	30	29.4

### 2.2 Data collection instruments

Data were collected using three instruments developed by the researchers:

1. A demographic characteristics questionnaire,
2. A post-resuscitation care practice questionnaire,
3. A post-resuscitation care knowledge test developed according to the 2021 ERC guidelines.

The preliminary knowledge assessment form initially consisted of 16 items. The draft questionnaire was evaluated by two emergency medicine specialists, one assessment and evaluation expert, and one faculty member from an emergency medical services program. Experts were asked to evaluate each item as “appropriate,” “inappropriate,” or “appropriate after revision,” and to provide recommendations when necessary.

Inter-rater agreement among expert evaluations was assessed using the Krippendorff alpha coefficient (8), which was calculated as 0.864, indicating high agreement. Following expert feedback, revisions were made to the questionnaire items. For items submitted again for expert review, the content validity ratio for each item was calculated using the Lawshe technique (9). At this stage, complete consistency was sought in expert opinions; 4 items where experts disagreed were removed from the questionnaire, and the final questionnaire was completed with 12 items that all experts deemed “appropriate” and had a Lawshe Content Validity Ratio (CVR) of 1.

### 2.3 Statistical analysis

Statistical analyses were performed using JAMOVI software (10, 11). Descriptive analyses were used to summarize demographic variables and scale scores. Continuous variables were expressed as mean ± standard deviation (SD), whereas categorical variables were presented as frequencies and percentages.

The Central Limit Theorem suggests that if the sample size is sufficiently large (*n* = 30+), the sampling distribution of means will be normally distributed regardless of the distribution of variables, and any deviation from normal distribution will not cause a major problem (12, 13). Skewness does not deviate significantly from normality in large samples. Positive kurtosis disappears in sample sizes greater than 100, and negative kurtosis disappears in sample sizes greater than 200 (12). Based on this information, the significance between the variables and the survey results was tested using *t*-tests and ANOVA comparison tests. Levene's test was used for the *t*-test, and Tukey's *post-hoc* test was used for the ANOVA test. Furthermore, looking at the kurtosis and skewness values, values between −1.96 and +1.96 indicate that the data does not show a significant deviation from normal distribution and that the normality assumption is met.

Additionally, two-way ANOVA analyses were conducted to evaluate interaction effects between:

- Workplace unit and years of professional experience,
- Professional title and time elapsed since the last advanced life support training,

on post-resuscitation knowledge scores.

A *p*-value of <0.05 was considered statistically significant.

Effect sizes were calculated for statistically significant group comparisons. Cohen's d values were interpreted as small (0.2), medium (0.5), and large (0.8) effects for t-tests (14). Eta squared (*η*^2^) values were interpreted as small (0.01), medium (0.06), and large (0.14) effects for ANOVA analyses (14, 15).

## 3 Results

A total of 318 emergency healthcare personnel were included in the study. Among all participants, the mean number of cardiac arrest cases receiving advanced life support per month was 4.90 ± 0.29. This value was significantly higher among emergency department personnel (8.65 ± 0.76) compared with prehospital EMS personnel (3.27 ± 0.16).

The mean transfer time of patients achieving ROSC from the prehospital setting to the emergency department was 20.35 ± 0.93 min. In addition, the mean transfer time from the emergency department to the intensive care unit was 95.26 ± 11.73 min.

Chi-square analysis demonstrated no statistically significant relationship between the working units of ambulance and emergency department healthcare personnel and their responses to the post-CPR care attitude questionnaire items (*p* > .05 for all items). The distribution of responses to the post-CPR care attitude questionnaire is presented in Table 2.

**Table 2:** Distribution of responses to the post-CPR care attitude survey.

Consider a patient who has achieved ROSC after resuscitation. Please answer Yes or No to each question below.		EMS Personnel (*n* = 216)	ED Personnel (*n* = 102)	χ^2^/p
		n (%)	n (%)	
1-If there is hemodynamic instability and/or ECG findings, I would consider the possibility of Acute Coronary Syndrome and would consider transfer to a center with angiography capabilities.	Yes	171 (79.2%)	81 (79.4%)	0.003/0.960
	No	45 (20.8%)	21 (20.6%)	
2-Even without ST elevation, I would consider transferring the patient to a center with angiography capabilities, suspecting Acute Coronary Syndrome.	Yes	69 (31.9%)	30 (29.4%)	0.038/0.845
	No	147 (68.1%)	72 (70.6%)	
3-Therapeutic hypothermia (targeted temperature management) should be applied.	Yes	195 (90.3%)	99 (97.1%)	0.019/0.891
	No	21 (9.7%)	3 (2.9%)	
4-I will inquire about the availability of an intensive care unit for the patient's transfer.	Yes	126 (58.3%)	84 (82.4%)	0.008/0.928
	No	90 (41.7%)	18 (17.6%)	
5-I will inquire about the availability of a palliative care unit for the transfer.	Yes	36 (16.7%)	18 (17.6%)	0.011/0.918
	No	180 (56.6%)	84 (82.4%)	
6-I routinely perform 12-lead ECGs.	Yes	141 (65.3%)	93 (91.2%)	0.000/0.988
	No	75 (34.7%)	9 (8.8%)	
7-I measure blood sugar levels.	Yes	213 (98.6%)	96 (94.1%)	0.007/0.935
	No	3 (1.4%9	6 (5.9%)	
8-I monitor urine output.	Yes	63 (29.2%)	93 (91.2%)	0.000/1.000
	No	153 (70.8%)	9 (8.8%)	
9-I will evaluate the arrhythmias by performing an ECG and continue with the treatment.	Yes	210 (97.2%)	99 (97.1%)	0.007/1.000
	No	6 (2.8%)	3 (2.9%)	
10-I begin treatment by focusing on the underlying causes (4H/4 T).	Yes	210 (97.2%)	99 (97.1%)	0.007/1.000
	No	6 (2.8%)	3 (2.9%)	

Among both EMS and ED personnel, the highest proportion of “yes” responses was observed for the following practices:

- Measuring blood glucose levels after ROSC,
- Evaluating cardiac rhythm using electrocardiography (ECG);
- Continuing treatment by focusing on reversible causes of cardiac arrest.

In contrast, the lowest proportion of positive responses was observed for:

- Evaluating suitability for transfer to palliative care units,
- Considering transfer to a coronary angiography-capable center despite the absence of ST-segment elevation.

### 3.1 Monitoring practices following ROSC

Among study participants, 66% reported monitoring vital signs every 5 min after ROSC, while 63.2% reported reassessing ECG findings, consciousness level, and airway status at the same interval. Additionally, 61.3% stated that they monitored SpO_2_, end-tidal carbon dioxide (ETCO_2_), and capillary refill time every 5 min.

No statistically significant difference was observed between EMS and ED personnel regarding the frequency of vital sign monitoring following ROSC (*χ*^2^ = 2.926, *p* = 0.232). However, significant differences were identified in:

- The frequency of reassessment of ECG, consciousness, and airway status (*χ*^2^ = 6.440, *p* = 0.040);
- The frequency of perfusion monitoring using SpO_2_, ETCO₂, and capillary refill time (*χ*^2^ = 8.021, *p* = 0.046).

Table 3 shows the frequency of evaluating patients by EMS and ED personnel after ROSC.

**Table 3:** Frequency of evaluating patients who ROSC.

After ROSC, how often do you perform the following assessments?	Once	At 10-minute intervals	At 5-minute intervals	At 1-minute intervals	χ^2^/p
1-Vital-sign monitoring					
EMS Personnel (*n* = 216)	0	45 (20.8%)	147 (68.1%)	24 (11.1%)	2.926/0.232
ED Personnel (*n* = 102)	0	30 (29.4%)	63 (61.8%)	9 (8.8%)	
2-Patient reassessment (ECG findings, level of consciousness, and airway status)					
EMS Personnel (*n* = 216)	0 ^a^	27 (12.5%) ^a^	135 (62.5%) ^a^	54 (25%) ^a^	6.440/0.040
ED Personnel (*n* = 102)	0 ^a^	21 (20.6%) ^a^	66 (64.7%) ^a^	15 (14.7%) ^b^	
3-Oxygenation and ventilation monitoring (SpO₂ and ETCO₂)					
EMS Personnel (*n* = 216)	3 (1.4%) ^a^	36 (16.7%) ^a^	138 (63.9%) ^a^	39 (18.1%) ^a^	8.021/0.046
ED Personnel (*n* = 102)	0 ^a^	30 (29.4%) ^b^	57 (55.9%) ^a^	15 (14.7%) ^a^	

### 3.2 Post-CPR knowledge scores

The post-CPR knowledge test was scored out of 100. The mean knowledge score among participants was 50.08 ± 13.78, indicating a moderate level of knowledge regarding post-resuscitation care.

The questions with the lowest correct response rates were:

- Seizure management after cardiac arrest (9.4%),
- Target blood glucose levels following cardiac arrest (12.3%).

The highest correct response rates were observed for:

- Indications for emergency coronary angiography in patients with ST-segment elevation myocardial infarction (92.5%),
- Minimum hourly urine output targets after ROSC (83%).

**Figure 1:**
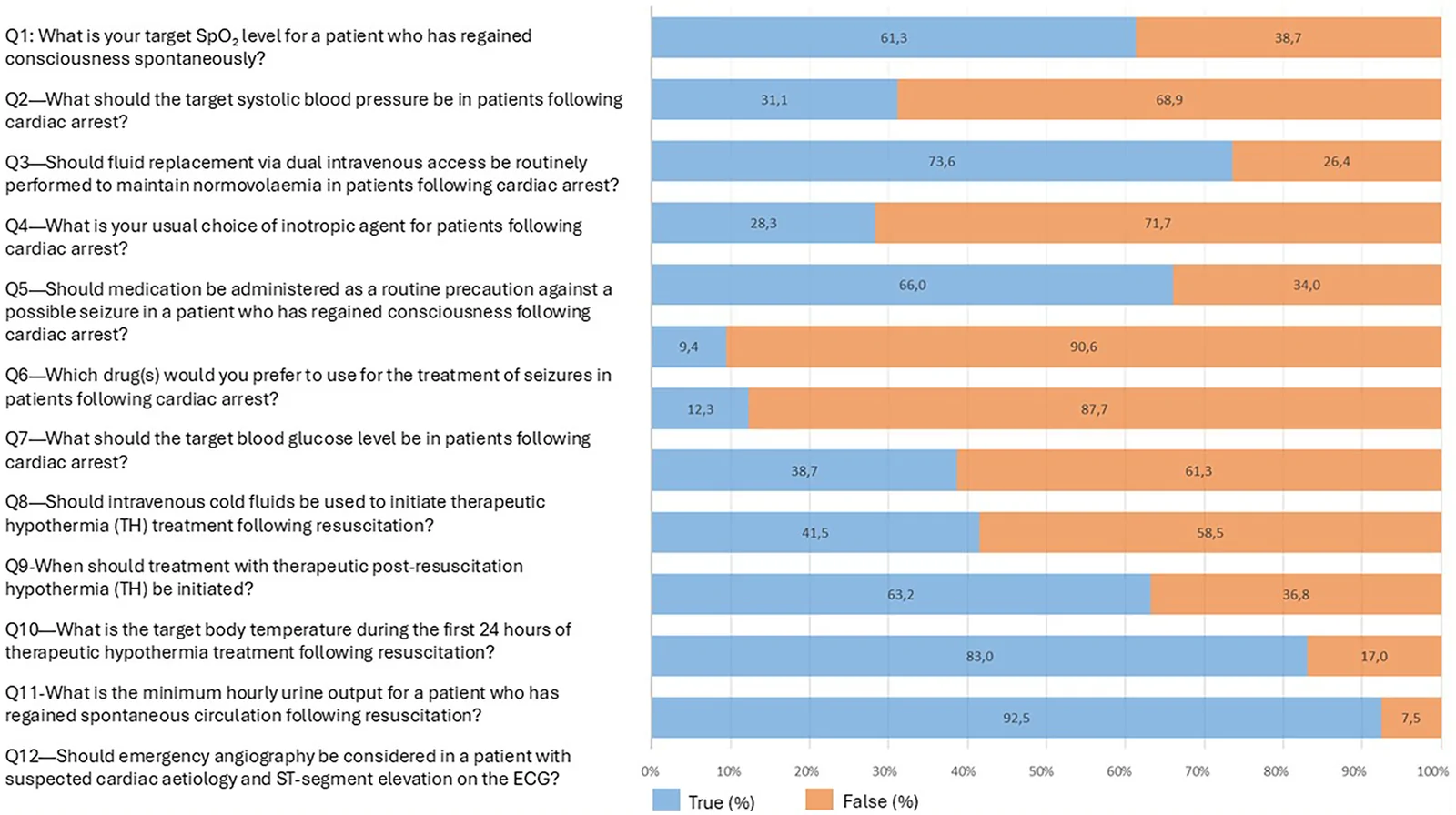
Distribution of correct and incorrect answers to post-CPR knowledge test questions.

The distribution of correct and incorrect responses is shown in Figure 1.

### 3.3 Comparison of knowledge scores according to demographic variables

Significant differences were observed according to workplace setting. ED personnel demonstrated significantly higher knowledge scores than EMS personnel (56.86 ± 13.84 vs. 46.87 ± 12.56, *p* < 0.001). Workplace setting showed a large effect size on post-CPR knowledge scores (Cohen's d = 0.769) (Table 4).

**Table 4:** Comparison of post-CPR knowledge scores by work department.

Groups	Variables	Post-CPR knowledge scores			
		$\bar{X}\pm \mathrm{ss}$	*t*/p	Significant Difference	Effect Size
Department	(1) ED Personnel	56.86 ± 13.84	6.401/ < .001	1 > 2	Cohen's d 0.769
	(2) EMS Personnel	46.87 ± 12.56			

No statistically significant differences were identified in post-CPR knowledge scores regarding gender or adherence to current guidelines (*p* > 0.05). When personnel working in emergency medical ambulances and emergency departments were evaluated separately, male emergency department personnel demonstrated significantly higher knowledge scores than their female counterparts with respect to the gender variable (*t* = 1.988, *p* = 0.049, *η*^2^ = 0.556). In addition, among emergency medical ambulance personnel, those who reported following current guidelines had significantly higher knowledge scores than those who did not, with respect to the variable of adherence to current guidelines (*t* = 2.539, *p* = 0.012, *η*^2^ = 0.449). These variables have a moderate effect size on knowledge scores.

A significant association was found between the total post-CPR care knowledge scores of emergency healthcare personnel and title (F = 5.067, *p* = 0.002), professional experience (F = 5.083, *p* < .001), and the time elapsed since the last advanced life support training (F = 3.621, *p* = 0.013). Regarding title, Emergency Medical Technicians (EMTs) had significantly lower mean knowledge scores than physicians and paramedics. Subgroup analyses of emergency ambulance and emergency department personnel also revealed significant differences according to title. Among emergency ambulance personnel, paramedics scored significantly higher than technicians (F = 6.648, *p* = 0.010), while among emergency department personnel, paramedics achieved significantly higher scores than the other professional groups (F = 14.554, *p* = 0.010). The effect size of title on post-CPR knowledge level was moderate (*η*^2^ = 0.031–0.046).

Significant differences were also observed according to years of professional experience. Participants with 11–20 years of experience had significantly lower mean scores than those with 0–1, 2–5, and 6–10 years of experience (F = 5.083, *p* < .001). Years of professional experience demonstrated a moderate effect on post-CPR knowledge levels (*η*^2^ = 0.061). Among emergency department personnel, participants with 6–10 years of experience had significantly higher scores than those with 21 years or more of experience (F = 5.849, *p* < 0.001). Similarly, among emergency ambulance personnel, those with 2–5 years of experience scored significantly higher than those with 11–20 years of experience (F = 6.691, *p* < 0.001).

A significant difference was also observed according to the time elapsed since the most recent advanced life support training. Participants who had received training within the previous year demonstrated significantly higher mean scores than those whose training had occurred five or more years earlier (F = 3.621, *p* = 0.013). The effect size of this variable on post-CPR knowledge level was small (*η*^2^ = 0.033). Among emergency ambulance personnel, participants whose most recent training had been completed within the previous year achieved significantly higher scores than those whose training had occurred more than five years earlier (F = 3.136, *p* = 0.026). In contrast, among emergency department personnel, participants who had received training within the previous year had significantly lower mean scores than those whose most recent training had occurred 3–5 years earlier (F = 3.448, *p* = 0.020).

The results of the comparisons of post-CPR knowledge levels according to demographic characteristics are presented in Table 5.

**Table 5:** Results of the comparison test of post-CPR knowledge scores according to demographic variables.

Groups	Variables	Post-CPR knowledge scores		
		Total	EMS Personnel	ED Personnel
		$\bar{X}\pm \mathrm{ss}$	$\bar{X}\pm \mathrm{ss}$	$\bar{X}\pm \mathrm{ss}$
Gender	(1) Female	49.99. ± 13.89	46.21 ± 12.54	55.75 ± 13.94
	(2) Male	50.25 ± 13.59	47.91 ± 12.60	63.33 ± 11.70
	*t*/p	0.151/0.880	0.972/0.332	1.988/0.049
	Significant Difference	–	–	1 < 2
	Effect Size (Cohen's d)	–	–	d:0.556
Following Current Guidelines	(1) Yes	50.20 ± 13.41	47.88 ± 12.17	55.90 ± 14.63
	(2) No	49.64 ± 15.15	42.30 ± 13.43	59.16 ± 11.65
	*t*/p	0.300/0.764	2.539/0.012	1.085/0.280
	Significant Difference	–	1 > 2	–
	Effect Size (Cohen's d)	–	d:0.449	–
Title	(1) Physician	57.14 ± 10.63	–	57.14 ± 10.63
	(2) Paramedic	51.59 ± 12.39	48.780 ± 10.05	70.83 ± 9.58
	(3) Emergency Medical Technician	46.43 ± 15.41	44.354 ± 14.96	62.50 ± 7.54
	(4) Other (Health Officer/Nurse)	50.49 ± 13.38	–	50.49 ± 13.38
	*F*/p	5.067/0.002	6.748/0.010	14.554/ < .001
	Significant Difference	3 < 1, 2	2 > 3	2 > 4,3,1
	Effect Size (Eta Square)	*η*²: 0.046	*η*²: 0.031	*η*²: 0.308
Professional experience	(1) 0–1 Years	53.05 ± 9.90	51.89 ± 7.69	56.25 ± 14.60
	(2) 2–5 Years	55.15 ± 11.42	55.86 ± 12.41	52.38 ± 6.30
	(3) 6–10 Years	54.38 ± 14.63	47.37 ± 11.31	67.06 ± 11.02
	(4) 11–20 Years	47.11 ± 13.41	43.96 ± 12.69	57.64 ± 10.03
	(5) 21 Years and Over	48.72 ± 15.48	44.44 ± 11.02	50.01 ± 16.52
	*F*/p	5.083/ < .001	6.691/ < .001	5.849/ < .001
	Significant Difference	1,2,3 > 4	2 > 4	3 > 5
	Effect Size (Eta Square)	η²= 0.061	η²= 0.113	η²= 0.194
Time has Passed Since the Last Advanced Life Support Training	(1) Less than 1 year	52.58 ± 14.11	50.69 ± 9.42	53.92 ± 16.61
	(2) 1–2 Years	50.33 ± 15.47	47.22 ± 16.82	58.33 ± 6.46
	(3) 3–5 Years	50.49 ± 13.15	47.32 ± 11.21	65.28 ± 11.52
	(4) More than 5 years	44.91 ± 10.54	42.26 ± 9.81	54.16 ± 13.85
	*F*/p	3.621/0.013	3.136/0.026	3.448/0.020
	Significant Difference	1 > 4	1 > 4	3 > 1
	Effect Size (Eta Square)	η²= 0.033	η²= 0.042	η²= 0.095

Descriptive statistics are presented as mean ± standard deviation. *p*-values shown in bold were considered statistically significant (*p* < 0.05). $\bar{X}$: Mean, sd: Standard deviation.

### 3.4 Two-Way ANOVA analyses

A statistically significant interaction effect was found between workplace environment and professional experience on knowledge scores (*p* < 0.001). Interaction analysis revealed that the pattern of knowledge variation among experience categories differed between emergency medical services and emergency department personnel. In each workplace environment, knowledge scores varied according to years of professional experience, but the direction and magnitude of these differences were not consistent across environments. These results indicate that the relationship between years of professional experience and post-CPR knowledge level differs between ambulance personnel and emergency department personnel.

Additionally, a significant interaction was identified between professional title and time elapsed since the last advanced life support training (*p* = 0.002). Physicians who had received advanced life support training within the previous 3–5 years demonstrated significantly higher scores than:

- EMTs trained 1–2 years earlier,
- EMTs trained more than 5 years earlier,
- Paramedics trained 3–5 years earlier,
- Paramedics trained more than 5 years earlier.

Furthermore, paramedics who had received training within the previous 1–2 years demonstrated significantly higher scores than EMTs within the same training interval.

This interaction demonstrated a moderate effect size (*η*^2^ = 0.087).

The results of the two-way ANOVA analyses are presented in Table 6.

**Table 6:** Results of the two-way ANOVA test.

Source	Sum of Squares	sd	Mean of Squares	F	p	Eta Square
Professional experience * Department	2,831.12	4	707.78	4.77	<0.001	0.058
Title * Time has Passed Since the Last Advanced Life Support Training	4,321.42	8	540.18	3.22	0.002	0.087

## 4 Discussion

In this study, the knowledge levels and clinical practice approaches regarding post-resuscitation care among emergency healthcare personnel working in different provinces of Türkiye were evaluated. The findings revealed that emergency healthcare staff generally possess a moderate level of knowledge regarding post-resuscitation care; however, significant gaps were identified in certain critical areas. Nevertheless, the moderate score obtained indicates that the level of knowledge regarding post-resuscitation care is insufficient. In addition, workplace setting, professional title, years of experience, and recency of advanced life support training were found to significantly influence post-resuscitation knowledge levels.

Participants reported performing advanced life support in an average of 4.90 cases of cardiac arrest per month. This frequency was significantly higher among emergency department staff compared to prehospital EMS personnel. This difference is thought to result from the central role of emergency departments in cardiac arrest management, the fact that post-EMS follow-up is performed by emergency departments, and the higher risk of cardiac arrest among critically ill patients treated in emergency departments (16, 17).

The mean transfer time of patients achieving ROSC from the prehospital setting to the emergency department was approximately 20 min, whereas transfer from the emergency department to intensive care units required approximately 95 min. Early transfer to advanced care units following ROSC is considered a major determinant of survival and neurological outcomes after cardiac arrest (18). Current European Resuscitation Council (ERC) and American Heart Association (AHA) guidelines recommend that ROSC patients should be transferred as rapidly as possible to centers capable of advanced monitoring, targeted temperature management, and organ support therapies (19, 20) Rapid transportation and continuity of care within the “chain of survival” are essential components of effective post-resuscitation management (21). Similarly, timely intensive care unit admission allows implementation of advanced hemodynamic monitoring, neurological assessment, and targeted temperature management strategies, all of which are associated with improved outcomes after cardiac arrest (22–25). The present study, the most frequently reported post-resuscitation practices included blood glucose monitoring, ECG rhythm assessment, and evaluation of reversible causes after ROSC. These findings suggest that emergency healthcare personnel demonstrate relatively high awareness regarding the fundamental clinical components of post-resuscitation care. Current ERC guidelines emphasize metabolic evaluation, rhythm monitoring, and identification of reversible etiologies as core components of post-cardiac arrest management (19, 26).

However, participants demonstrated lower awareness regarding transfer planning for coronary angiography-capable centers in patients without ST-segment elevation and assessment for palliative care referral. These findings suggest deficiencies in advanced post-resuscitation decision-making processes. Contemporary ERC guidelines emphasize that early coronary angiography may improve survival and neurological outcomes even in selected patients without ST-segment elevation, particularly when a cardiac etiology is suspected (19, 25, 26).

Approximately two-thirds of participants reported reassessing vital signs, airway status, ECG findings, oxygenation, perfusion, and neurological status every 5 min following ROSC. Such close monitoring is consistent with guidelines recommendations emphasizing continuous assessment of hemodynamic stability, ventilation, oxygenation, and neurological function during the early post-resuscitation period (19, 26, 27).

Nevertheless, approximately one-third of participants reported less frequent monitoring practices, indicating heterogeneity in clinical implementation. This variability highlights the need for standardized educational interventions and protocol-driven monitoring strategies based on current evidence and resuscitation guidelines (27, 28).

The mean post-resuscitation knowledge score was approximately 50 out of 100, indicating moderate knowledge levels among emergency healthcare personnel. Nevertheless, the moderate score obtained indicates that the level of knowledge regarding post-resuscitation care is insufficient. Similar findings have been reported in previous studies evaluating CPR and post-CPR care knowledge among healthcare professionals (17). The lowest correct response rates were observed in questions related to seizure management and target blood glucose levels after cardiac arrest. These findings suggest that neurological and metabolic management remain relatively weak domains in post-CPR care education.

Participants achieved the highest correct response rates in questions related to emergency coronary angiography in ST-segment elevation myocardial infarction and urine output monitoring after ROSC. These results suggest that emergency healthcare personnel may be more confident in hemodynamic and cardiovascular monitoring than in neurological or metabolic management. Previous studies similarly demonstrated that healthcare providers tend to perform better in cardiovascular and hemodynamic aspects of post-CPR care while demonstrating deficiencies in neurological and metabolic management (17, 29, 30).

No significant association was identified between knowledge scores and either sex or self-reported guidelines follow-up. This finding may indicate that passive awareness of guidelines alone is insufficient for maintaining practical knowledge and competencies. Previous literature has demonstrated rapid deterioration of resuscitation knowledge and skills over time (31, 32). Suggesting that conventional training programs may not adequately sustain long-term competency (28). Consequently, simulation-based, scenario-oriented, and high-frequency refresher training programs may represent more effective strategies for maintaining post-resuscitation care competencies (17, 28, 29). This association between professional experience and post-CPR knowledge levels may be explained by the mandatory refresher training programs (Adult Advanced Life Support, Pediatric Advanced Life Support, and Trauma Advanced Life Support) provided to emergency ambulance personnel at five-year intervals. Emergency service personnel participate in these trainings voluntarily.

In the resuscitation medicine literature, it has been reported that healthcare professionals' levels of knowledge regarding CPR are associated not only with the length of professional experience but also with job title, clinical role, frequency of resuscitation practice, and participation in up-to-date training programs. Basic and advanced life support knowledge levels can vary significantly among different professional groups, such as physicians, nurses, paramedics, and EMTs. Access to standardized training, familiarity with algorithms, and active participation in resuscitation practices in clinical settings are more influential factors in these differences than professional hierarchy (33). Resuscitation guidelines also highlight the potential for a decline in knowledge and skills and recommend that healthcare professionals providing advanced life support participate in structured and up-to-date training programs at regular intervals (34, 35).

The relationship between years of professional experience and resuscitation knowledge is not linear. While some studies report that clinical awareness and confidence in practice increase with experience, other studies show that long-term clinical experience alone is not sufficient to ensure knowledge of current guidelines. In particular, regularly updated resuscitation algorithms, the increasing complexity of post-ROSC care, and the loss of knowledge and skills that occurs as the time since training elapses suggest that up-to-date training plays an even more decisive role than professional seniority (35, 36).

In this context, considering professional experience alone as a measure of competence in assessing resuscitation knowledge is inadequate.

The findings of our study are largely consistent with the existing literature. The significant differences in knowledge levels we observed across job titles indicate that knowledge of post-ROSC care is not evenly distributed among the groups. The fact that paramedics have higher knowledge scores compared to some other groups may be related to this occupational group's more frequent involvement in resuscitation processes in prehospital emergency medical services, their more regular exposure to algorithm-based training, and their participation in mandatory refresher training at specific intervals (17, 29). This suggests that paramedics' knowledge advantage does not stem solely from their title; rather, it can be explained by their job description, clinical exposure, and the continuity of structured training.

Another notable finding in our study is that participants with longer professional experience (11–20 years) had significantly lower scores on the post-ROSC care knowledge test compared to less experienced groups. Although this result may seem, at first glance, to contradict the common expectation that experience would increase knowledge levels, it is consistent with studies reporting that knowledge and skills in resuscitation training can diminish over time (37, 38). Long-term clinical experience does not always mean that current guidelines are followed or that advanced life support training has been recently updated. Particularly in a field involving multifaceted decision-making processes—such as post-ROSC care, which encompasses oxygenation targets, hemodynamic optimization, neurological assessment, seizure management, coronary reperfusion strategies, and intensive care planning—access to up-to-date information and repeated training are of critical importance (39).

The fact that participants who received advanced life support training within the past year scored significantly higher than those who received training 3–5 years or more ago is consistent with the emphasis on continuing education in the literature. This finding supports the notion that knowledge of post-CPR care is not a static acquisition but a dynamic competency that must be regularly updated. The recommendations of ERC and the American Heart Association regarding resuscitation training also emphasize that training should not be limited to a one-time certification approach but should be supported by periodic refresher courses, simulation-based learning, teamwork, structured feedback, and competency assessment (35, 36).

The most notable commonality between our study and the literature is that professional title and the recency of training constitute decisive factors in post-ROSC care knowledge. Unlike the literature to some extent, our study found that greater professional experience is not always associated with a higher level of knowledge. However, this difference is significant not because it contradicts current evidence, but because it concretely demonstrates that knowledge may become outdated among experienced personnel who have drifted away from current training.

The study results showed that emergency department personnel demonstrated significantly higher knowledge scores than EMS personnel. This difference may reflect greater exposure to in-hospital cardiac arrest cases, more frequent participation in advanced resuscitation procedures, and increased access to continuing education programs among emergency department staff (17, 29, 30). Similarly, previous studies reported that formal training significantly improves peri-arrest and cardiac arrest rhythm recognition among prehospital emergency personnel (16). These findings support the need for more intensive simulation-based and recurrent educational programs targeting EMS personnel.

Professional title, years of experience, and recency of advanced life support training also significantly influenced post-resuscitation knowledge levels. Emergency medical technicians demonstrated lower knowledge scores than physicians and paramedics, supporting previous findings that higher-level professional roles are associated with stronger theoretical and practical competencies (29). Interestingly, participants with 11–20 years of experience demonstrated lower scores than less experienced personnel. This finding may reflect knowledge attrition over time and insufficient exposure to updated guideline-based training.

Participants who had received advanced life support training within the previous year demonstrated significantly higher knowledge scores than those trained more than five years earlier. This finding further emphasizes the importance of regular refresher education. Previous studies have shown that simulation-based practical training improves long-term retention of resuscitation skills and enhances clinical performance (40, 41). In particular, low-dose, high-frequency educational approaches and short-interval refresher courses have been reported to significantly improve both CPR quality and cardiac arrest survival outcomes (42–44).

In light of the study's findings, there is currently no consensus regarding knowledge and practice of cardiac arrest management among pre-hospital and emergency department personnel in Turkey. It is clear that, in order to achieve standardization, training programs must be standardized in accordance with current guidelines and repeated at regular intervals.

In addition to simulation-based education, technology-assisted learning strategies may further enhance long-term knowledge retention and clinical adaptability. Emerging educational tools such as virtual reality, augmented reality, gamified learning systems, smartphone-based educational applications, and serious gaming platforms are increasingly recognized as valuable supplements to traditional resuscitation training (45–49). These innovative approaches may be particularly beneficial for healthcare professionals working in high-intensity clinical environments by facilitating continuous learning independent of time and location constraints.

The study's findings provide important insights into areas where greater standardisation is required in post-CPR care processes. Although participants demonstrated a relatively adequate level of knowledge regarding haemodynamic monitoring and cardiovascular management following the ROSC and reported adherence to guidelines, significant gaps in the standard of care were identified from a neurological and metabolic perspective, particularly concerning seizure management and glycaemic control. These findings suggest that future standardisation efforts must go beyond cardiovascular stabilisation and place greater emphasis on the comprehensive management of post-cardiac arrest syndrome. Based on the observed knowledge gaps, a practical framework for improvement could include: (1) training based on a standardised guidelines focusing on post-cardiac arrest neurological and metabolic management; (2) periodic advanced life support refresher training, particularly for pre-hospital personnel; (3) the implementation of standardised post-resuscitation care protocols in pre-hospital, emergency department and intensive care settings; and (4) multidisciplinary collaboration to ensure continuity of care from the ROSC through to admission to intensive care. Such measures may help to reduce variations in practice and improve adherence to current post-CPR care guidelines.

## 5 Limitations

Several limitations of this study should be considered when interpreting the findings. First, the study was conducted using a cross-sectional design; therefore, causal relationships between variables could not be established. Second, the data were obtained through self-reported questionnaires, which may have introduced recall bias and response bias. Participants may have overestimated or underestimated their actual clinical practices and knowledge levels.

Third, although participants were recruited from 17 different provinces across Türkiye, the study population was limited to volunteers who responded through social media-based survey distribution. Consequently, selection bias may have affected the representativeness of the sample, and the findings may not be fully generalizable to all emergency healthcare personnel.

Within the scope of this study, the occupational title reflects the required level of formal education for each occupational group; however, data regarding additional individual qualifications were not collected.

Furthermore, the study evaluated clinical approaches based on theoretical knowledge and self-report rather than direct observation of real-life clinical performance. Therefore, actual bedside competencies and adherence to post-resuscitation care protocols could not be objectively assessed. Another limitation is that the study did not evaluate institutional differences such as hospital type, educational infrastructure, availability of simulation training, or local post-resuscitation care protocols; all of which can affect knowledge levels and clinical practices. This study did not evaluate institutional characteristics such as level of care (secondary and tertiary hospitals), availability of an intensive care unit, or participation in simulation-based training. Consequently, the observed differences should be interpreted with caution. Future studies should incorporate institutional and educational variables to better explain their impact on resuscitation knowledge and adherence to current guidelines.

The internal consistency reliability coefficient (KR-20) of the achievement test used in this study was calculated to be below acceptable thresholds (0.57). Item analysis revealed that certain questions fell into extreme ranges (either excessively difficult or excessively easy) relative to the knowledge level of the sample group (*N* = 318), resulting in negative values in the corrected item-total correlations. In knowledge-based assessments, such deviations in item discrimination and difficulty indices can artificially suppress the reliability coefficient by restricting the variance. To prevent compromising the content validity and the instrument's capacity to represent the target universe, these items were retained in the analysis, thereby preserving the original structure of the test. While this situation is acknowledged as a limitation of the current study, it is recommended that future research recalibrate the difficulty levels of the test items according to the target audience and conduct more in-depth distractor analyses.

Finally, rapidly evolving international resuscitation guidelines may influence post-resuscitation care practices over time. As a result, periodic reassessment studies are needed to evaluate the sustainability of knowledge retention and the impact of updated educational interventions on clinical practice.

## 6 Conclusion

This study demonstrated that emergency healthcare personnel possess a moderate level of knowledge regarding post-CPR care, with significant differences according to workplace setting, professional role, years of experience, and recency of advanced life support training. Emergency department personnel exhibited higher knowledge scores than prehospital EMS personnel, whereas important deficiencies were identified particularly in neurological and metabolic aspects of post-cardiac arrest management.

Although participants showed relatively high awareness regarding hemodynamic monitoring, rhythm evaluation, and reversible causes after ROSC, gaps remained in advanced clinical decision-making processes such as post-arrest coronary angiography assessment and palliative care planning. In addition, variability in monitoring frequency and clinical practice approaches suggests a lack of standardization in post-CPR care implementation.

These findings emphasize the need for structured, guideline-based, and simulation-oriented educational programs aimed at improving post-CPR care competencies among emergency healthcare personnel. Regular refresher training, low-dose high-frequency educational strategies, and technology-assisted learning approaches may contribute to enhancing both knowledge retention and clinical performance. Strengthening post-CPR care education and standardizing clinical practice may ultimately improve survival and neurological outcomes in patients with cardiac arrest.

## Data Availability

The raw data supporting the conclusions of this article will be made available by the authors, without undue reservation.
